# Haptically Guided Grasping. fMRI Shows Right-Hemisphere Parietal Stimulus Encoding, and Bilateral Dorso-Ventral Parietal Gradients of Object- and Action-Related Processing during Grasp Execution

**DOI:** 10.3389/fnhum.2015.00691

**Published:** 2016-01-05

**Authors:** Mattia Marangon, Agnieszka Kubiak, Gregory Króliczak

**Affiliations:** Action and Cognition Laboratory, Department of Social Sciences, Institute of Psychology, Adam Mickiewicz University in PoznańPoznań, Poland

**Keywords:** haptic exploration, encoding bias, action planning, grasp execution, complex objects, dorsal stream

## Abstract

The neural bases of haptically-guided grasp planning and execution are largely unknown, especially for stimuli having no visual representations. Therefore, we used functional magnetic resonance imaging (fMRI) to monitor brain activity during haptic exploration of novel 3D complex objects, subsequent grasp planning, and the execution of the pre-planned grasps. Haptic object exploration, involving extraction of shape, orientation, and length of the to-be-grasped targets, was associated with the fronto-parietal, temporo-occipital, and insular cortex activity. Yet, only the anterior divisions of the posterior parietal cortex (PPC) of the right hemisphere were significantly more engaged in exploration of complex objects (vs. simple control disks). None of these regions were re-recruited during the planning phase. Even more surprisingly, the left-hemisphere intraparietal, temporal, and occipital areas that were significantly invoked for grasp planning did not show sensitivity to object features. Finally, grasp execution, involving the re-recruitment of the critical right-hemisphere PPC clusters, was also significantly associated with two kinds of bilateral parieto-frontal processes. The first represents transformations of grasp-relevant target features and is linked to the dorso-dorsal (lateral and medial) parieto-frontal networks. The second monitors grasp kinematics and belongs to the ventro-dorsal networks. Indeed, signal modulations associated with these distinct functions follow dorso-ventral gradients, with left aIPS showing significant sensitivity to both target features and the characteristics of the required grasp. Thus, our results from the haptic domain are consistent with the notion that the parietal processing for action guidance reflects primarily transformations from object-related to effector-related coding, and these mechanisms are rather independent of sensory input modality.

## Introduction

When searching a key in a deep pocket, or reaching for an electric torch in a drawer right after an evening power outage, our fingers are used to actively explore the encountered shapes to find the desired target. Yet, when there is only a single, and unobstructed goal object with a familiar size and/or structure, the hand—even though directed somewhat “blindly”—may already be suitably open and even rotated in anticipation for grasping the expected target. Such skilled actions are possible in the absence of direct vision because the control of manual tasks in the sighted person is under such conditions likely mediated by the visually-encoded properties of objects processed in the ventral *perceptual* stream (Goodale and Milner, 1992; Milner and Goodale, 2008; see also Króliczak et al., 2008; Singhal et al., 2013; cf. Ungerleider and Mishkin, 1982). Of course, purely sensorimotor and/or kinesthetic information (e.g., Fiehler et al., 2008) must be also incorporated in the functioning of the dorsal *action* stream (Goodale and Milner, 1992) for the guidance of such motor skills (For a recent review on the contributions of visual and haptic information to reaching and grasping see Stone and Gonzalez, 2015; see also a review on somatosensory processes involved in perception and action by Dijkerman and de Haan, 2007).

It is not known, though, whether or not action guidance would rely on similar circuits if confronted with completely unfamiliar objects or their shapes that had never been encoded with the use of vision—a situation a person who loses sight later in life would be confronted with. On the one hand, there is compelling evidence that when object shape information is first acquired exclusively by active touch (haptic exploration) its encoding is associated not only with the dorsal, superior parietal lobule activity (Binkofski et al., 1999a) but can also invoke ventral stream regions, such as the ventro-lateral extents of the occipital lobe typically associated with visual tasks (James et al., 2002; see also Amedi et al., 2001, 2002). On the other hand, the *haptic* parallels to dissociated visual processing of objects for different tasks (e.g., Ungerleider and Mishkin, 1982; cf. Goodale and Milner, 1992; see also Rizzolatti and Matelli, 2003; Binkofski and Buxbaum, 2013) are limited. That is, despite evidence that haptic object recognition (what an object is) vs. its localization (where it is positioned) is also mediated by relatively independent mechanisms, both of these skills have been shown to invoke the dorsal-stream regions. In fact, it has been demonstrated that there is somewhat greater inferior parietal lobule contribution to haptic object recognition, and bilateral superior parietal lobule involvement in tactile object localization (Reed et al., 2005; see also Reed et al., 2004). Therefore, the pathways underlying haptically-driven action guidance (Dijkerman and de Haan, 2007) may differ markedly from those originally proposed for visually-guided actions. That is, the superior parietal cortex may underlie encoding of object properties for the control of actions directed toward these objects (cf. Jäncke et al., 2001; Fiehler et al., 2008), whereas the more ventral pathways, including secondary somatosensory cortex and terminating in the insula, may play a greater role in object recognition (see also James et al., 2007).

Here, we investigated the neural underpinning of haptically-guided grasping directed at objects never seen before. To this end, functional magnetic resonance imaging (fMRI) was used to measure the blood oxygen level dependent (BOLD) signal changes associated with exploration of the shape and orientation of novel objects, the subsequent grasp planning, and the actual execution of grasping movements directed at these objects. As such, all the tasks performed in this experiment were based entirely on the haptically acquired information. Not only were we interested in testing for any analogies to visually guided performance of grasping but we were also interested to get to know the patterns of brain activity that would emerge during the preparatory phases, ultimately leading to the grasping of the target objects. Based on previous studies on delayed manual actions (Fiehler et al., 2011; Singhal et al., 2013), we hypothesized that the areas involved in object shape, size and orientation encoding—i.e., engaged during haptic exploration—would be later invoked for object grasping. We also assumed that grasping of the more complex objects (vs. much simpler circular disks) could reveal not only the involvement of the superior parietal lobule (Binkofski et al., 1999a) but also some ventral stream, and/or insular cortex contribution to the task (James et al., 2002; Dijkerman and de Haan, 2007). Finally, we hypothesized that if the encoded object shape information is stored over a brief delay period, its reactivation during the planning phase may invoke re-recruitment of regions anterior to the ones that would be engaged during the grasping task (cf. Valyear et al., 2007; Singhal et al., 2013). That is, anterior vs. posterior activity gradients were expected within regions contributing to planning vs. execution of haptically-guided actions.

## Methods

### Participants

Ten *University of Oregon* students and postdoctoral fellows with no history of neurological or psychiatric disorders (four females; mean age = 28.1, *SD* = 5.2) took part in this study after giving written informed consent. All of them were right-handed as measured by the Edinburgh Handedness Inventory (Oldfield, 1971), had normal or corrected-to-normal visual acuity (important mainly for reading instructions and just one control task), and they were all compensated financially for their time. The local Ethics Committee, and the Bio-Ethics Committee at Poznan University of Medical Sciences, approved the experimental protocols, which conformed to the WMA Declaration of Helsinki.

### Familiarization phase

There was a short practice that took place just before the study proper, with objects that were not a part of the experimental set. Participants were told that their task is to explore the novel objects in order to find their axes of elongation because they will be later asked to grasp these objects. Everybody was encouraged to explore the targets carefully to be absolutely sure how they should be grasped, and it typically took the whole exploration time to perform this task for the majority of complex objects. As to simple circular disks, participants were asked to explore them for the whole task interval (so that any difference in brain activity should not be due to lack of exploratory movements in this simpler task, but due to clear differences in object shape processing). There was no specific instruction about how the grasp planning should be performed. As to grasping the complex objects, participants were explicitly told to grasp them along their long axes. Circular shapes, conversely, were to be grasped in the most convenient way. Importantly, participants were asked not to correct for any grip imprecision, and were instructed not to lift the objects (off the surface to which they were attached).

### Stimuli and procedure

**Figure 1:**
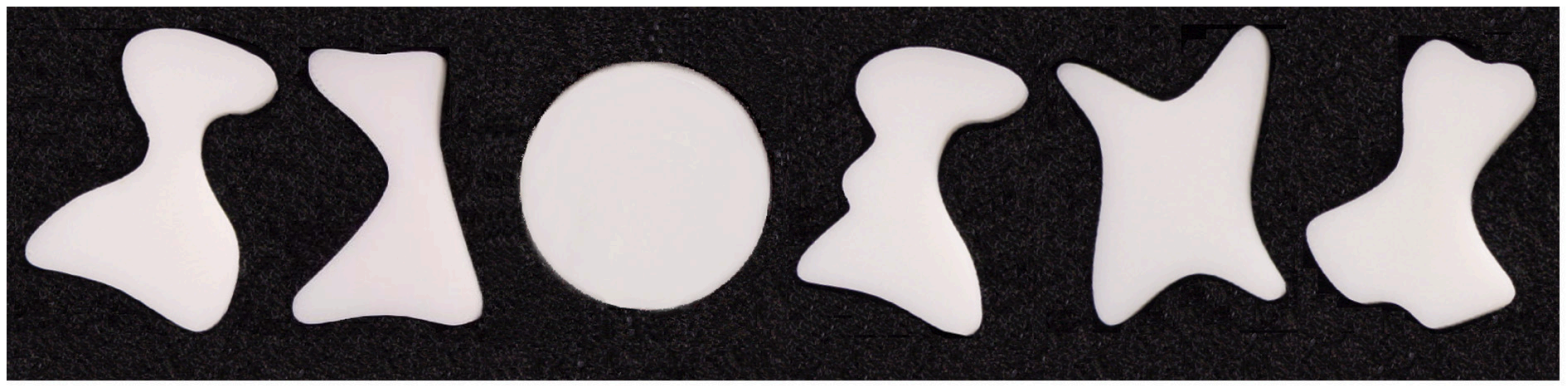
**Examples of three-dimensional stimuli used in the experiment**. Only one object was presented at a given time. Participants neither saw these stimuli before, nor during the study proper (i.e., no visual feedback was ever provided during any of the tasks). Although the orientation of the objects varied, their location remained the same. Upon their haptic exploration, and subsequent planning, participants grasped these targets with a precision (pincer) grip, using the index finger and thumb. The complex objects were always grasped along their longer axes, whereas the circular disks were typically grasped with the most comfortable grip.

The experimental stimuli consisted of 32 three-dimensional objects of different shapes and sizes, and most of them were merely larger versions of the stimuli used earlier by Króliczak et al. (2008). Made of white translucent plastic, these objects had a constant depth (of 0.6 cm), but varied in length (between 3.4 and 4.6 cm), and width (typically between 2.6 and 3.6 cm, although the narrower part of objects was close to 0.7 cm). The examples of stimuli used are shown in Figure 1, and all of them, their order, and orientations are shown in Supplementary Figure 1. Arranged pseudorandomly into four (4) sets of eight objects of different shapes, orientations (with an equal number rotated slightly leftward and rightward), and/or to some degree also sizes, with each set including two circular control disks (4 cm in diameter), they were attached to four *Velcro* strips. The separate strips were then placed centrally on, and presented with, a custom made MR-compatible device somewhat resembling the “*Grasparatus*” created and used in the *Culham Lab* (e.g., Króliczak et al., 2008).

Each consecutive trial consisted of a different object, but each object in a given set was eventually presented three times, thus resulting in 24 trials per run. The four sets of objects were changed pseudorandomly between each of the five consecutive runs for each participant. This means that one of the object sets (a random one, but most often the one that was used in the first run) was presented for the second time in the 5th and last run. Thus, each participant received a different, pseudorandom order of target objects (by manipulating the order of object sets). They were attached to the *grasparatus's* drum, which was located above the participants' hips. Notably, for the person lying in the MRI scanner, the stimuli were within the reach of the hand, but could be neither seen directly nor via the mirror (which actually reflected instructions from the screen located behind the scanner). Similarly, the participants did not have any visual feedback of their hands.

**Figure 2:**
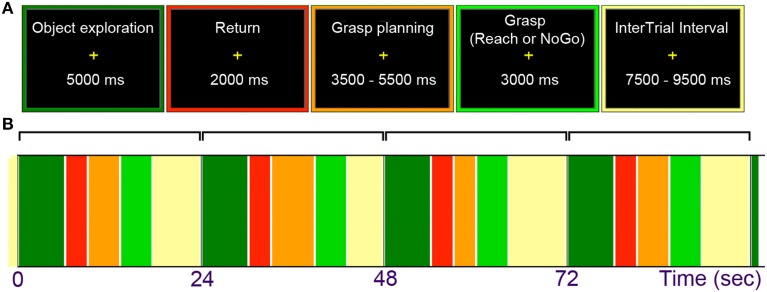
**(A)** General trial structure and possible timing of its events. **(B)** An example of initial trial layout. Haptic exploration began with an “Explore” cue and lasted for 5 s. Upon a return of the hand to the starting position, grasp planning was initiated by the “Plan” cue and lasted through a variable interval of 3.5, 4.5, or 5.5 s. Grasp execution was triggered with a “Grasp” cue (on 50% of trials), and reaching actions with a “Reach” cue (on 25% of trials in which participants touched the objects with the knuckles). On the remaining (25%) of “NoGo” trials, triggered by the “Wait” cue, participants were to abort a response and waited for the final “Rest” cue. This cue began a variable inter-trial interval lasting 7.5, 8.5, or 9.5 s for all trial types.

All the manual tasks in the main study of this project were performed with the dominant right hand, whose initial position was indicated by a custom-made start key placed near the belly button. A participant was first asked to explore the presented object for 5 s (starting with an “EXPLORE” cue), and then to move the hand back to press the key within the subsequent 2 s (a period clearly marked with a “RETURN” cue). Next, during a variable interval of 3.5, 4.5, or 5.5 s, the task was to plan a grasping movement of the just explored object (with the beginning of this task indicated with a “PLAN” cue). Subsequently, 50% of the trials involved the execution of the pre-planned grasp (triggered by a “GRASP” cue) wherein a complex object was always grasped along its longer axis, and a simple circular disk was grasped with the most comfortable grip and hand orientation. In 25% of trials the task was simply to reach toward an object and touch it with the knuckles (the “REACH” cue), and in the remaining 25% of trials a participant was asked to withhold a planned response (upon hearing the “WAIT” cue, which given the cue that followed effectively meant “no-go”). Each task concluded with a “REST” cue, resulting in an inter-trial interval that varied between 7.5 and 9.5 s (starting from the beginning of the cue). Upon completion of a given task by the participant, the experimenter rotated the drum manually to present the next object. The drum rotation typically followed the “REST” cue, which was easy to time because the experimenter could also hear all the cues via headphones. Given the adopted duration and variability of events within trials, a single run typically lasted just over 9 min. Trial structure and timing is shown in Figure 2.

Initially, we intended to present all the task cues (e.g., explore, return, grasp, etc.) auditorily via the MR-compatible headphones, with the duration of each cue set to 750 ms. However, given that during pilot testing a volunteer complained about distortions in auditory signal, to make sure that all the cues can be easily understood, in addition to fixing sound quality we also decided to present visual cues for 1.5 s each. This was done with a white Tahoma Regular font on a black background, in capital letters, size 100, subtending ca. 10 × 2° when projected on a screen behind the scanner bore, and viewed via a mirror from a distance of ~70 cm. The onsets of the cues were synchronized. This manipulation resembles naturally occurring situations wherein we may hear a request for action and see an accompanying visual signal (e.g., gesture) that strengthens its clarity, but we actually do not see the target of the to-be-performed response.

Testing was carried out in a darkened room. Although the “commands” were displayed visually, the grasping task was guided exclusively based on information obtained with haptic exploration a few seconds before, including the variable time interval for grasp planning. Because no visual feedback was ever provided during task performance, its execution (due to task novelty, i.e., little practice with the task and novel stimuli) seemed quite difficult at first. To make grasping actions a bit easier, despite changes in orientation, the objects were presented in the same central location.

### Additional localizer scans

All the 10 participants were also tested in at least two different functional localizer runs (Some of the participants agreed to perform a given localizer scan twice).

The first functional localizer served to identify the brain area known as *aIPS*, and the tactile-visual subdivision of the lateral occipital cortex, dubbed LOtv. As the first acronym implies, the aIPS is located anteriorly in the intraparietal sulcus (typically on its lateral bank, near or at the intersection with the postcentral gyrus), and it has been linked to the guidance of grasping movements (e.g., Binkofski et al., 1998; Culham et al., 2003; Króliczak et al., 2007; cf. Gallivan et al., 2009; Monaco et al., 2011). As the second acronym implies, the LOtv is a multimodal area, located anteriorly, and more inferiorly, to area MT+, at the junction of the ascending limb of the inferior temporal gyrus, and the lateral occipital sulcus (Amedi et al., 2002). Participants were asked to search for and explore small toy plastic objects, such as animals, tools, and other man made gadgets, placed among irregular pebble-like or more regular cube-like plastic shapes in the bags attached to the wrists of their hands. The task was to find a meaningful shape with the tips of the fingers, categorize it if possible, and continue searching for further toys resembling common objects. There were 12 blocks of the exploration task, each lasting 16 s, interleaved with 12 blocks of 16-s rest periods, during which the fingers were kept still, but touched the shapes, regardless of whether they were meaningful or not. The order of blocks was counterbalanced across participants.

The second functional localizer served to identify the brain area known as *LO cortex*. Located on the lateral extent of the occipital lobe this area is typically defined by viewing intact vs. scrambled objects (e.g., Kanwisher et al., 1997; see also Ferber et al., 2003; Large et al., 2005). Typically, this object-selective area—implicated in the bottom-up analyses of visual shapes—is located right behind (but may also partly overlap with) the motion-selective area MT+ (cf. Kourtzi and Kanwisher, 2000; see also Dumoulin et al., 2000).

It was the only task in this study when participants actually looked at visual images of objects and their scrambled counterparts, and it was always run last. In addition to common household objects and tools, participants were also shown computer generated novel objects (used earlier by Harman et al., 1999; Króliczak et al., 2003), as well as the silhouettes of the previously explored shapes. Indeed, it was actually the first time when the haptically experienced shapes were also encoded visually. Ten (10) different objects, with one random repeated, each separated by a 150-ms mask composed of thin intersecting parallel (horizontal and vertical) lines, were shown in six blocks lasting 12 s, separated either by six blocks of 10 scrambled objects (one random repeated) which were also separated by the 150 ms mask, or by six blocks of rest periods with a fixation point. There were two blocks with visual images of common items, two blocks with novel objects, and two blocks with the silhouettes of the haptically experienced shapes. The same number of blocks was used for the presentation of their scrambled counterparts. Participants performed a one back task wherein they were to indicate with a button press the appearance of the repeated object, or the repeated scrambled pattern.

Because exploratory finger movements may not only be associated with the engagement of LOtv, but also motion sensitive regions (Kourtzi and Kanwisher, 2000; Amedi et al., 2002; see also Dumoulin et al., 2000), for a more in-depth interpretation of the results it has been necessary for us to know the location of the motion-selective area MT+. It was established by two multi-localizer scans from a different cohort of 21 right-handed participants of similar age (11 females). Areas sensitive to two kinds of visual motion, and to the control of two kinds of hand movements were identified. The right and left hands were always tested separately, and typically on 2 consecutive days, whereas the visual stimuli remained basically the same. These stimuli typically consisted of superimposed radial, and concentric gratings, similar to the ones used by Culham et al. (1999), rotating either clockwise, and/or counter-clockwise in three different 14-s blocks (24 steps of 15° rotation per block) or contracting and/or expanding, again in three different 14-s blocks (4 consecutive steps of 1.7° forward or backward movement, changing position 24 times per block). During hand movement tasks, participants were asked to either rotate their wrist in four steps during the three different 14-s blocks (clockwise and counter-clockwise in a pace similar to the previously seen visual changes), or to reach out and move the arm back, again in four steps during the three different 14-s blocks (back and forth, in a pace similar to the contraction/expansion of the visual image). All the conditions were pseudorandomized, with one of the two visual conditions being always presented first when it comes to task blocks, and were supplemented with six (6) 14-s blocks of passive viewing of stationary radial, and/or concentric control gratings, and additional six (6) 14-s rest periods, with a fixation dot in the middle of the screen.

### MRI procedures

In the main experiment, and two localizer scans that immediately followed, a Siemens Allegra 3T MRI system (Siemens, Erlangen, Germany) equipped with echo planar imaging (EPI) capabilities, with a 12-channel phased array transmit/receive head coil, was used for data acquisition at the *Lewis Center for NeuroImaging* at the University of Oregon. Supplemental localizer scans were acquired at the Nencki Institute of Experimental Biology in Warsaw using a Siemens TRIO 3T Scanner with a 32-Channel Head coil, and very similar imaging parameters. Functional volumes were collected using a T2^*^-weighted, segmented gradient-echo echo planar imaging (time to echo/time to repetition [TE/TR] = 30/2000 ms, flip angle [FA] = 80°, voxel size = 3.125 × 3.125 mm; field of view = 384 mm). Each volume was made up of 32 contiguous slices of 3.5-mm thickness. The initial first four volumes in each scan series were discarded. In the main “*haptic*” experiment of this project, each participant performed five functional runs composed of 275 volumes each. AIPS localizer scans involved the acquisition of 196 volumes per run, during the LO localizer scans only 156 volumes were obtained, whereas during the MT+/hand-movement multi-localizer 225 volumes were acquired on each day. High-resolution anatomical scans were collected using a 3D T1-weighted MPRAGE sequence (TE/TR = 4.38/2500 ms; FA = 8.0°, 176 contiguous axial slices, thickness = 1.0 mm, voxel size = 1.0 × 1.0 mm; field of view = 256 mm). Siemen's Auto Align Scout and True FISP sequences were executed for each participant before data collection to ensure that slices were prescribed in exactly the same positions across runs. DICOM image files were converted to FSL NIfTI format using the software called MRIConvert (http://lcni.uoregon.edu/\simjolinda/MRIConvert/).

### fMRI data analyses

Data analyses were performed using the FMRIB Software Library (FSL) version 5.0.6 (Jenkinson et al., 2012). The initial preprocessing steps involved: the use of *Brain Extraction Tool* (BET) for non-brain tissue removal (Smith, 2002), the application of motion correction MCFLIRT algorithm (Jenkinson et al., 2002), spatial smoothing with a Gaussian kernel of full width half magnitude (FWHM) = 8 mm, and high-pass temporal filtering with a cutoff = 50 s. In the functional data from the main (*haptic*) experiment, Siemens EPI-navigated *prospective motion correction* algorithm, followed by automatic retrospective re-acquisition, was applied during data collection, and the use of MCFLIRT was no longer required. (In fact, as indicated before, the use of additional motion correction algorithm would in such a case be detrimental; Króliczak, 2013).

Whole brain (*voxelwise*) analyses were performed using FSL's *fMRI Expert Analysis Tool* (FEAT), part of FSL (Jenkinson et al., 2012). At the first level, each fMRI run was analyzed separately, with each condition modeled with a canonical hemodynamic response function (double-gamma HRF). Nine predictors, in the FSL software referred to as *Explanatory Variables* (EVs) were used, including two separate EVs—for complex and simple objects, respectively—for the three main conditions, i.e., *Exploration* (of complex, and simple objects), *Grasp Planning* (for complex, and simple objects), and *Grasp Execution* (for complex, and simple objects), as well as one EV for *Reaching* trials, one for *NoGo* trials, and finally one for “Rest” periods (i.e., the variable longer intervals between consecutive trials). Temporal derivatives for each explanatory variable were automatically created as additional regressors in order to correct for timing discrepancies (e.g., to correct for slice timing alignment).

Except for the *Grasp Planning* activity, which was modeled as the 3.5-s period beginning with the onset of the instructional cue (i.e., presented visually for 1.5 s, though aurally only for 0.75 s) and lasting through the end of the shortest (2.0 s) delay interval (as in Króliczak and Frey, 2009; and Króliczak et al., 2011; see also Figure 2), Exploration, Grasp, Reach, and NoGo conditions, as well as the baseline “Rest intervals” were modeled for their entire durations. Note also that the variable delay introduced between the *grasp planning* and *execution* phases substantially reduced the temporal coupling of the two phases, thus enabling an easier deconvolution of the signal from these disparate tasks (e.g., Króliczak and Frey, 2009; cf. Marangon et al., 2011.) The non-modeled *Return* intervals following exploration played the same role (here: clearly separating the exploration and planning-related signals), and together with the non-modeled “tails” of delay intervals for planning, as conditions of no interest, contributed to the calculation of mean activity in the run (the so-called *implicit baseline*). While testing for the main effects of tasks vs. explicitly-defined baseline activity, i.e., exploration vs. rest, plan vs. rest, etc., regardless of object type involved, greater weights were actually put on activity related to more complex tasks (i.e., +0.75 for complex objects, and +0.25 for simple disks, vs. −1 for rest). Of course, during testing for simple main effects of each task, and in all direct contrasts between the conditions, the balanced weighting was applied to each of the contrasted conditions.

The resulting first-level contrasts of parameter estimates (COPEs) served as inputs to the second-level analyses (within subjects, across individual runs) using a Fixed Effects model. The resulting second-level COPEs were then used as inputs to the third-level analyses (across participants), performed using a *mixed-effects model, with the random-effects components of variance* estimated with the default FSL's procedure, the so-called *FLAME stage 1* (Beckmann et al., 2003). For the two critical contrasts, i.e., *Grasping Complex object* vs. *Reaching*, and *Grasping Complex* vs. *Simple objects*, additional analyses were also run with the more time consuming but therefore more robust *FLAME stage* 1+2 procedure (Beckmann et al., 2003). The outcomes from the whole brain (voxelwise) analyses are depicted in figures showing only significant clusters of signal modulations, typically in the form of increased brain activity. Inclusive contrast masking procedure was applied to identify areas significantly activated across two comparisons. In all the renderings, Z-statistic (Gaussianized T/F) images were thresholded with the use of *Z* > 2.3 and a corrected cluster significance threshold of *P* = 0.05 (Worsley, 2001). These are the default settings in the FSL's FEAT fMRI analysis tool, where significance level for each cluster is first estimated from Gaussian Random Field theory, then compared with the cluster probability threshold, and corrected accordingly. FSL *LInear Registration Tool* (FLIRT; as described by Jenkinson and Smith, 2001) was used to implement registration of functional images to high-resolution and standard space images (from the Montreal Neurological Institute [MNI-152] 1 mm brain template).

Anatomical localization of clusters with significant brain activity was always verified by manual comparison with an atlas (Damasio, 2005), and by projecting and visualizing these maps using the standard mapping algorithm implemented in the Caret software (http://www.nitrc.org/projects/caret/), where the group statistical imaging maps can be conveniently overlaid onto a population-average, landmark- and surface-based (PALS) human brain atlas (Van Essen, 2005). Overlays of activity were obtained with the Caret “convert metric to RGB” function, followed by additional adjustments and mixing of the overlaid colors in the three surface renderings.

### Region of interest (ROI) analyses

A total of eight ROIs were selected and/or defined based on voxelwise group results from the main study, the outcomes from the two functional localizer scans, and a combination of thereof with the *Juelich cytoarchitectonic maps* and/or anatomical regions from the *Harvard-Oxford probabilistic atlas* included in the FSL package. Indeed, the additional use of the probabilistic maps also helped verifying the anatomical locations of our ROIs. In order to ensure extraction of separate clusters in a given brain region, the probabilistic maps were thresholded at (i.e., zeroed below) the 30% of their lower probability tails. (This way, for example, the middle frontal gyrus ROI did not include any of the voxels belonging functionally to the premotor cortex of the precentral gyrus.) Notably, although the selection of separate functional ROIs is very easy to perform manually using the “*paint*” tools in the FSLview package, the application of probabilistic atlases to extract ROI “masks” (volumes covering distinct regions) from group-average contrast activity also allows for an objective demarcation of clusters which are connected, and the removal of voxels at the borders with white matter. If necessary, additional localizers from the on-going projects in the lab were used for a comparison and/or clarification.

The primary goal of the ROI analyses was to determine the relative contribution of each selected area to all major studied *tasks* (exploration, planning, grasping), including task difficulty related to *object type* (complex, simple). To this end, a 3 (task) × 2 (object type) ANOVA was run on brain activity from their respective contrasts vs. the resting baseline, including the removal of signal related to instruction processing (i.e., exploration of complex objects vs. rest and instruction processing, exploration of simple objects vs. rest and instruction processing, grasp planning for complex objects vs. rest and instruction processing, etc.). The most common level of significance was adopted, i.e., α = 0.05. Where necessary, the required *post-hoc* tests were Bonferroni adjusted (marked as “Bf-p” to indicated that the *P*-value was corrected for multiple comparisons).

We focused only on left-hemisphere parietal and frontal areas which are typically linked to higher order manual skills (including action planning and execution; e.g., Frey et al., 2005; Króliczak and Frey, 2009; Jacobs et al., 2010; Marangon et al., 2011), and several ventral areas, such as MTG or LO expected to play a role in less rehearsed or delayed actions (e.g., Króliczak et al., 2007; Singhal et al., 2013).

## Results

### Haptic object exploration

**Figure 3:**
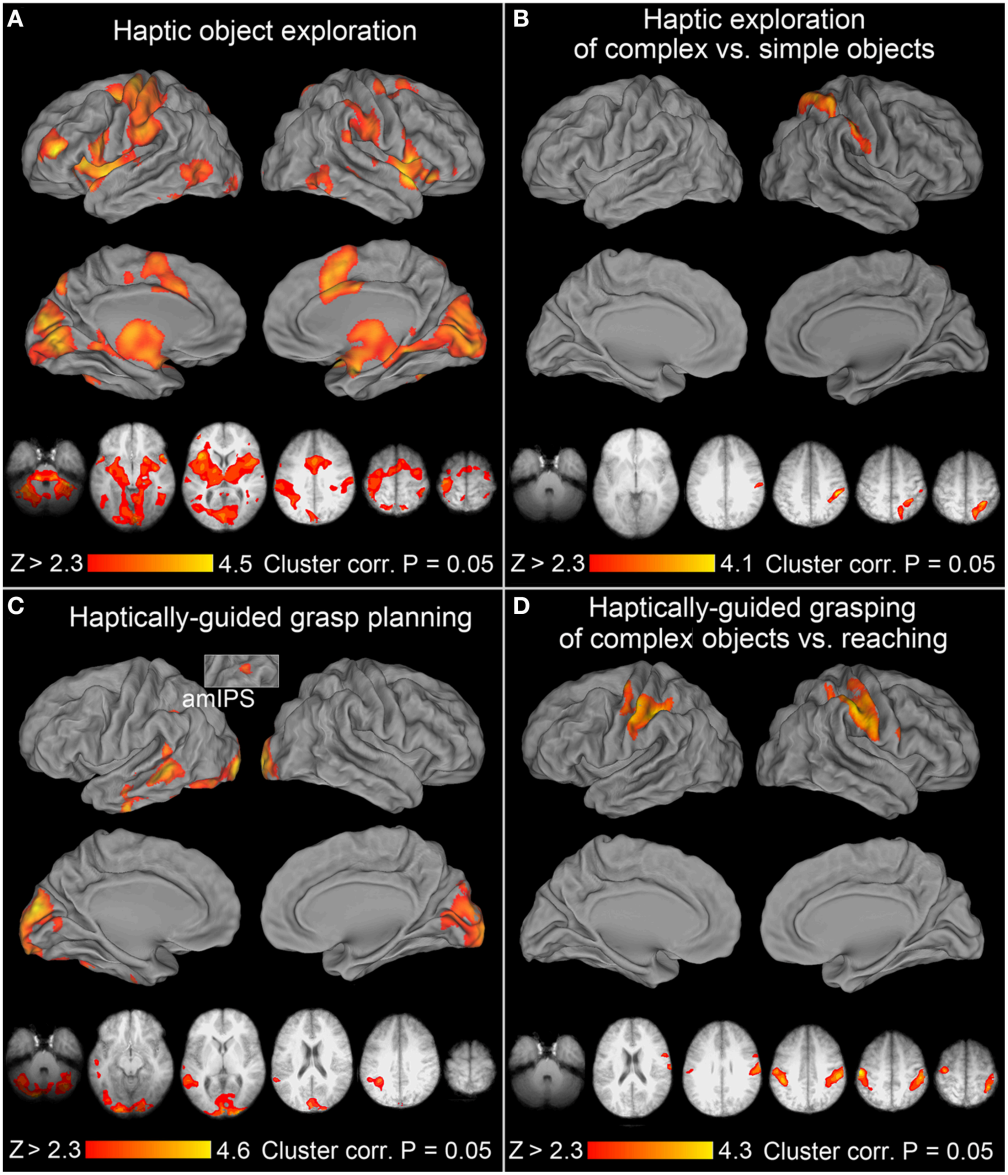
**Neural activity associated with haptic exploration, grasp planning, and grasp execution**. In all the panels of this, and subsequent, figure, group mean statistical parametric maps were thresholded at *Z* > 2.3, and a corrected clusterwise significance threshold of *P* = 0.05. The upper volumetric surface renderings in each of the panels illustrate significant group averages from the selected contrasts overlaid on the PALS atlas, whereas the lower axial slices illustrate this same activity in the most informative slices of an *average brain* obtained from all study participants' T1-weighted anatomical scans. All the images are displayed in neurological convention (i.e., right hemisphere is on the right), and are shown in hues corresponding to the color bars at the bottom of the panels. **(A)** *Haptic exploration*: there were significant increases of activity in all the major areas of *the praxis representation network* (PRN). They include the anterior supramarginal gyrus (aSMG), anterior intraparietal sulcus (aIPS), ventral premotor cortex (PMv), dorsal premotor cortex (PMd), supplementary motor areas (SMA complex), cingulate motor cortex (CMC), rostral middle frontal gyrus (rMFG), and the middle temporo-occipital cortex (TOC). The remaining clusters were observed in the early visual cortices (EVCs), superior parieto-occipital cortex (SPOC), including its anterior and dorsal subdivisions (adSPOC), primary and secondary somatosensory cortices (SI, SII), the thalamus, the insular cortex, and the neighboring superior temporal cortex. Except for the left sensorimotor cortex, most of the areas were engaged bilaterally. **(B)** *Haptic exploration of complex* vs. *simple objects*: the significant increases of activity were exclusively right lateralized and involved adSPOC, rostral superior parietal gyrus (rSPG), aIPS, aSMG, the fundus of the ventral postcentral gyrus (vPCG), and possibly SII. **(C)** *Haptically-guided grasp planning*: in addition to bilateral EVCs, extending into posterior fusiform gyrus (pFusG) on the left, the remaining activity was exclusively left lateralized and involved anterior-to-mid IPS (amIPS), posterior middle temporal gyrus (pMTG), caudal superior temporal gyrus (cSTG), and more anterior divisions of middle and inferior temporal gyri (aMTG and aITG). **(D)** *Haptically-guided grasping of complex objects* vs. *reaching toward them*: the significant modulations of activity involved aIPS, sensorimotor cortices, and a very small PMd cluster on the left, as well as SI, SII, aSMG, rSPG, and PMv on the right.

After accounting for instruction processing, and when compared to the resting baseline, the haptic exploration of target objects was associated with a bilateral engagement of both the parieto-frontal networks, and the occipito-temporo-insular networks (consistent with James et al., 2002; Dijkerman and de Haan, 2007). The contribution of the left hemisphere was greater for three reasons: (1) the lower-level sensorimotor activity was, due to the use of the right hand, almost exclusively left lateralized, (2) except for the subcortical and medial cortical structures, such as the pre-supplementary and the cingulate motor areas (pre-SMA, and CMA respectively), the clusters of activity were typically larger on the lateral surfaces of the left hemisphere, including aIPS, the anterior division of the supramarginal gyrus (aSMG), secondary somatosensory cortex (SII), and the ventral premotor cortex (PMv), and finally, (3) the very rostral subdivision of the middle frontal gyrus (rMFG) was engaged exclusively on the left. The clusters of significant activity revealed by this contrast are shown in the form of surface renderings, and in the most representative slices in Figure 3A.

Of note is the widespread activity on the medial surfaces in the striate and extrastriate areas of the occipital lobe (early visual cortices or EVCs, as in Singhal et al., 2013), and in the lateral temporo-occipital cortices (TOC), including its posterior division belonging to MT+ (as revealed by an overlap with voxels having >50% probability of belonging to the *cytoarchitechtonic* map of V5 from the Juelich atlas, and an MT+ localizer from our lab). Moreover, the more medial clusters were connected, via the parahippocampal gyrus, to the thalamic activity, which in turn was linked to the mid-to-anterior insular cortices, and closely related clusters in anterior divisions of the superior temporal gyri. In the left precuneus, the observed signal modulations overlapped with the antero-dorsal divisions of the superior parieto-occipital cortex (adSPOC, cf., Hutchison et al., 2015), whereas in the right precuneus they were more rostral and dorsal. Finally, there was a clear involvement of the dorsal premotor (PMd) cortex, although in the right hemisphere the activity extended more onto the superior frontal gyrus.

### Haptic exploration of complex vs. simple objects

Consistent with earlier studies (Binkofski et al., 1999a; Reed et al., 2005), the haptic exploration of complex objects (vs. simple circular disks) was associated with significant signal modulations in anterior divisions of the posterior parietal lobe, spanning both its superior and inferior lobules, including the rostral superior parietal gyrus (rSPG), aIPS and aSMG, but also extending slightly onto SII. Notably, this single, dorso-ventrally stretched cluster of activity was exclusively right lateralized. This effect is shown in Figure 3B, again on surface renderings and in the most representative slices.

### Haptic exploration of simple vs. complex objects

None of the areas from the parieto-frontal action network showed significantly greater activity in this contrast. Conversely, a widespread and often interrelated net of clusters resembling the default mode network was revealed. Because all these regions and/or most of their subdivisions were not even activated when compared to the resting baseline, the observed effects result primarily from greater inhibition of this network during a more difficult task. These findings are illustrated in Supplementary Figure 2 in the most representative slices.

### Grasp planning based on haptically obtained information

After the subtraction of signal related to instruction processing, and when compared to the resting baseline, the significant grasp planning activity was localized primarily to the occipital and temporal cortices, and mainly to the left hemisphere. Interestingly, there was also a relatively small cluster of activity observed in the left amIPS, and in the premotor cortex (also on the left). Yet, the premotor cluster was conspicuously extended into the white matter, and there were spurious signal modulations in the vicinity of the corpus callosum. For these reasons, this contrast was re-run in the brain mask deprived of white matter (including the corpus callosum itself). The premotor cluster turned out to be too small to reach significance threshold. The remaining significant cortical activity was unaffected by this reanalysis, and is shown in Figure 3C.

In addition to the bilateral involvement of EVCs, including the dorso-medial striate and extrastriate cortices, the planning-related activity—regardless of object type—was observed in the left ventro-lateral divisions of the occipital lobe, most likely including ventral visual area 4 (V4v), extending into the posterior fusiform gyrus (pFusG), and further into the occipito-temporal sulcus. The most conspicuous cluster was found in the posterior middle temporal gyrus (pMTG), although its caudal division extended dorsally (via the superior temporal sulcus) to the caudal superior temporal gyrus (cf. Glover et al., 2012). A smaller cluster of activity was also observed in the more anterior division of the inferior temporal sulcus and gyrus.

### Grasp planning for complex vs. simple objects (and vice versa)

None of the planning related significant activity was sensitive to object type because the contrast of planning the grasp for complex vs. simple objects was empty. The inverse contrast also revealed lack of significant differences with one exception: significantly weaker inhibition of the primary motor cortex on the left during the planning of simple grasps (cf. the exploration of simple vs. complex objects above).

### Haptically-guided grasp execution

Similarly to the exploration task, when compared to the resting baseline and with instruction processing accounted for, the haptically-guided grasping of objects was again associated with a bilateral engagement of both the parieto-frontal networks, and the occipito-temporo-insular networks (with activity pattern quite similar to the one shown in Figure 3A for the exploration task). Interestingly, in addition to being more symmetrical, the activity was larger in its extent, and included regions that were not invoked during the exploration of objects, such as the superior temporal sulci and gyri (with their large bilateral involvement). Moreover, the left caudal intraparietal sulcus (cIPS), and the SPOC region were also clearly involved (cf. Gallivan et al., 2009; Monaco et al., 2015). However, when directly compared with activity from the exploration, neither of the parieto-occipital nor the temporo-occipital regions was more significantly engaged in grasping.

### Haptically-guided grasping vs. reaching

To enable comparisons with earlier studies on visually guided grasping, we first ran a balanced contrast of *Grasping [of Complex and Simple Objects]* > *Reaching*. This contrast was empty. However, since reaching always involved the presence of complex objects, and the participants did not know ahead of time that it was going to be a reaching trial, the reaching task was quite demanding (i.e., required the processing of object features and a change in cognitive decision/manual performance). Therefore, a more appropriate comparison would involve a balanced contrast between *Grasping of Complex Objects* vs. *Reaching toward Complex Objects*. This was indeed the case. In addition to the expected greater involvement of the sensorimotor cortex on the left, a widespread somatosensory (primary or SI, and SII) engagement on the right, this contrast also revealed a familiar contribution of left aIPS (and to a lesser degree its right hemisphere counterpart), as well as rSPG, aSMG, and PMv exclusively on the right. In fact, except for the missing bilateral PMd and left SPOC contribution, this dorsal stream activity was very similar to the one observed for grasping vs. reaching in a study by Króliczak et al. (2008). The observed significant clusters of activity are shown in Figure 3D.

### Haptically-guided grasping of complex vs. simple objects

**Figure 4:**
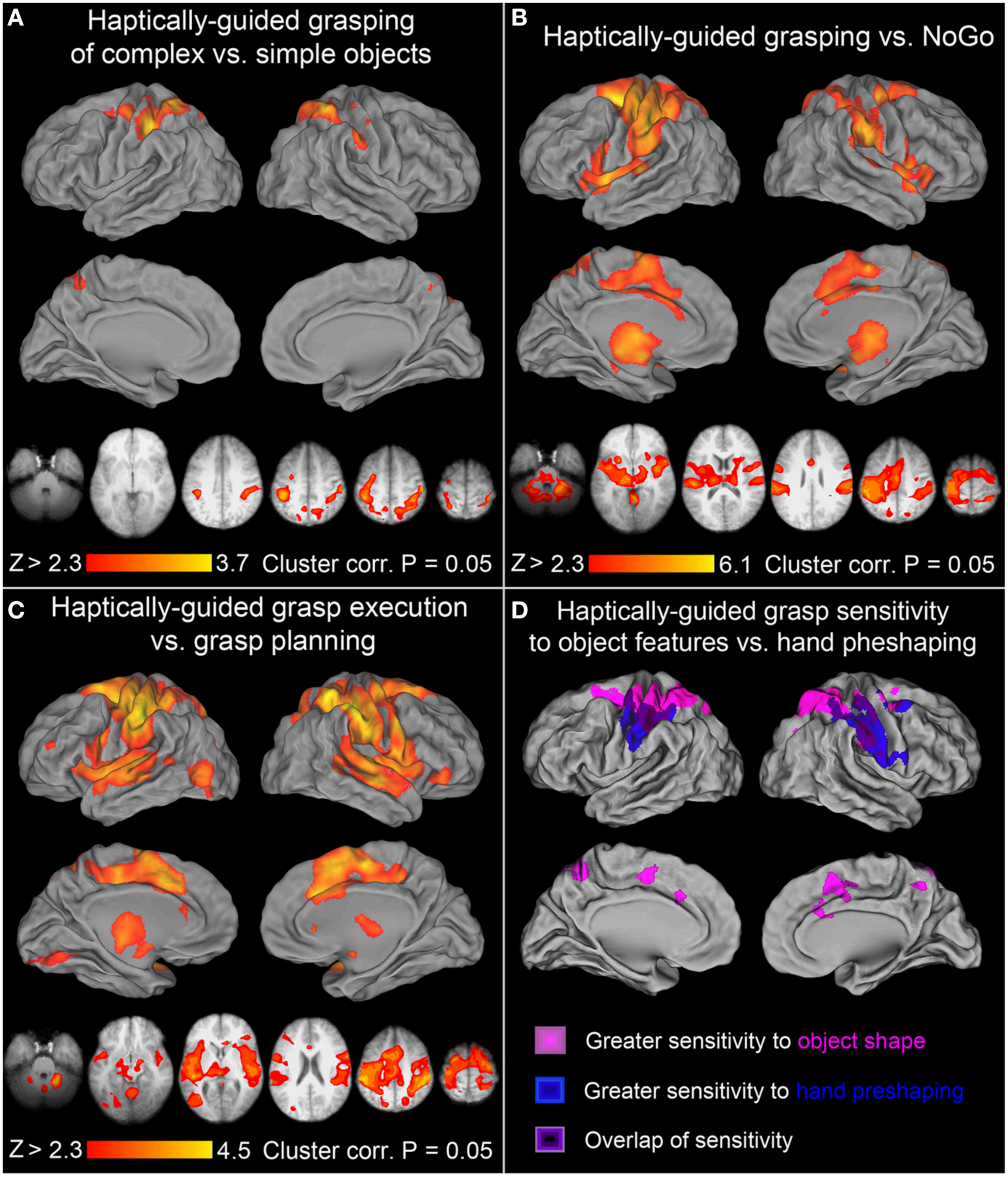
**Further contrasts and comparisons showing neural activity associated with haptically-guided grasping**. **(A)** *Grasping of complex* vs. *simple objects*: the significant clusters involved left aIPS, rSPG, adSPOC, PMd, and the sensorimotor cortex, whereas in the right hemisphere all the voxels sensitive to object features and involved in related processing (of complex vs. simple objects) were re-recruited, including adSPOC, rSPG, aIPS, aSMG, vPCG, and SI. **(B)** *Grasping* vs. *NoGo*: in addition to all the areas, or their subdivisions, significantly sensitive to object type [see **(A)**], this contrast also revealed bilateral involvement of SII, PMv, SMA complex, CMC, middle and anterior insular cortices and the neighboring STGs, as well as the thalamus, and caudate nucleus. **(C)** *Grasp execution* vs. *Grasp planning*: this contrast revealed significant involvement in of all the areas from the previous contrast, supplemented by a small contribution from left rMFG, the orbital division of the right inferior frontal gyrus, left EVCs, and the lateral occipital complex, encompassing its primarily visual lateral occipital division (LO), the multimodal tactile and visual division (LOtv), and the motion sensitive area MT+. As in panel **(C)**, these additional areas did not show much sensitivity to haptically processed object features that are of critical importance for haptically-guided grasping. **(D)** *The dorso-ventral gradients of grasp-related activity associated with different kinds of processing*: All the areas depicted in magentas represent grasp-relevant features of the unseen targets and belong to the dorso-dorsal (lateral, but also medial) parieto-frontal networks. All the areas illustrated in blues contribute to monitoring of hand pre-shaping or grasp kinematics, and belong to the ventro-dorsal networks. Finally, the areas depicted in violets, including left aIPS, show significant sensitivity to both grasp-relevant target features and the characteristics of the required grasp.

Another important effects were revealed by a balanced contrast of the two grasping conditions (namely, Grasping Complex > Grasping Simple objects), as it shows all the brain areas sensitive to critical object features during grasp performance and/or how they are translated into appropriate grip scaling. As it turns out, nearly all the right-hemisphere PPC voxels that were sensitive to object shape during their exploration (see Figure 3B) were now re-recruited for the grasping of these objects. This activity was accompanied by significant signal increases in the sensorimotor areas of the left hemisphere contributing to hand guidance. But even more importantly, left aIPS, and PMd, as well as voxels anterior to classically defined area SPOC, also showed sensitivity to object features during grasping. All significantly activated clusters during grasping of complex objects—i.e., showing sensitivity to object shape—are shown in Figure 4A. Of particular note is the contribution of the left superior (dorso-dorsal) parieto-frontal network, and left aIPS.

### Haptically-guided grasping of simple vs. complex objects

This contrast was empty. No area showed significantly greater modulation in this simpler task.

### Haptically-guided grasping vs. NoGo

All the nodes forming the bilateral parieto-frontal and temporo-insular networks involved in haptically-guided grasping—regardless of whether they are sensitive to object features or not—are revealed by a contrast of grasping vs. the NoGo condition. The obtained significant signal modulations are shown in Figure 4B. Although the network of areas has now expanded substantially, and includes bilateral parietal opercular and temporal clusters on the other side of the Sylvian Fissure, bilateral PMv, and on the medial surfaces SMA, pre-SMA, and the nearby CMA, none of the occipital or temporal regions involved in object processing was identified by this contrast. Importantly, neither the temporo-insular clusters nor, even more surprisingly, the bilateral PMv and SMA complex, showed substantial sensitivity to object type during grasping (see Figure 4A).

### Haptically-guided grasping vs. grasp planning

One of the most surprising outcomes so far has been the conspicuous absence of the LO cortex contribution to the haptically-guided grasping task in any of the direct contrasts between the major studied conditions, including “grasping vs. NoGo” (except for the comparisons of exploration, and grasping, vs. the resting baseline). Indeed, LO was not even involved in the planning of grasp (vs. the resting baseline) either. To shed some more light on this issue, we directly compared the grasp-execution phase with the grasp-planning phase. In this contrast, LO contribution has been revealed, in addition to the widespread differences in nearly all the areas mentioned thus far in the context of grasping, and exploration. This outcome is depicted in Figure 4C. It must be emphasized that, although this effect is not driven by the LO inhibition during grasp planning—but rather weak, non-significant fluctuations of activity around the resting baseline—its contribution to grasping is marginal at best. After all, consistently with earlier studies (e.g., Króliczak et al., 2007), the signal modulations observed in this area do not depend in any way on object type, and as such they cannot contribute directly to the control of grasping.

### Haptically-guided grasp planning vs. grasp execution

The contrast was nearly empty, except for a cluster of weaker inhibition observed bilaterally in the medial frontal cortex, which was not even invoked in a contrast of grasp planning vs. rest.

The anatomical locations of all the major clusters revealed in the contrasts described above, the MNI coordinates, as well as the statistical values of the peak voxels can be found in Table 1.

**Table 1:** **Major contrasts from the Main Haptic Experiment and the Localizer Scans**.

**Region**	**MNI coordinates**			**Peak value z-max**
	***x***	***y***	***z***	
**MAJOR CONTRASTS FROM THE MAIN HAPTIC EXPERIMENT**				
**A. Haptic object exploration vs. rest (*Z* > 2.3, *p* = 0.05 cluster corrected)**				
Right pre-Supplementary Motor Area (pre-SMA)	4	4	54	3.16
Right Cingulate Motor Area (CMA)	4	16	31	3.12
Left anterior Intraparietal Sulcus (aIPS)	–32	–49	29	3.90
Left anterior Supramarginal Gyrus (aSMG)	–56	–36	30	3.15
Left Secondary Somatosensory Cortex (SII)	–55	–23	9	3.05
Left dorsal Premotor Cortex (PMd)	–26	–11	51	3.22
Left ventral Premotor Cortex (PMv)	–57	10	23	3.18
Left rostral Middle Frontal Gyrus (rMFG)	–38	39	13	3.60
Posterior Calcarine Sulcus	0	–83	1	3.30
Left Lateral Temporal-Occipital Cortex (TOC)	–44	–70	2	2.77
Left posterior Middle Temporal Gyrus (pMTG)	–50	–55	–5	2.82
Left Precuneus	–3	–79	42	3.09
**B. Haptic exploration of complex vs. simple objects (*Z* > 2.3, *p* = 0.05 cluster corrected)**				
Right rostral Superior Parietal Gyrus (rSPG)	25	–59	61	3.70
Right anterior Intraparietal Sulcus (aIPS)	34	–45	53	3.42
Right anterior Supramarginal Gyrus (aSMG)	43	–34	41	4.00
Right Secondary Somatosensory Cortex (SII)	57	–19	28	2.93
**C. Grasp planning vs. rest (*Z* > 2.3, *p* = 0.05 cluster corrected)**				
Anterior Calcarine Sulcus	0	–88	9	3.80
Posterior Calcarine Sulcus	0	–100	3	3.06
Left anterior-to-mid Intraparietal Sulcus (amIPS)	–58	–39	–4	3.30
Left dorsal Premotor Cortex (PMd)	–34	–2	37	3.05
Left ventral Visual Area 4 (V4v)	–13	–93	–11	3.43
Left posterior Fusiform Gyrus (pFusG)	–50	–19	–30	3.37
Left posterior Middle Temporal Gyrus (pMTG)	–58	–48	4	3.35
**D. Haptically-guided grasp execution vs. rest (*Z* > 2.3, *p* = 0.05 cluster corrected)**				
Most of the areas and their coordinates are the same as in **A**. Haptic exploration vs. rest. Additional regions are listed below.				
Left Superior Temporal Gyrus (pSTG)	–63	–22	–1	3.23
Right Superior Temporal Gyrus (pSTG)	58	–21	–1	3.30
Left Caudal Intraparietal Sulcus (cIPS)	–43	–46	53	3.63
Left Superior Parieto-Occipital Cortex (SPOC)	–12	–79	45	3.06
**E. Haptically-guided Grasping vs. Reaching (*Z* > 2.3, *p* = 0.05 cluster corrected)**				
Left anterior Intraparietal Sulcus (aIPS)	–49	–37	37	3.13
Right rostral Superior Parietal Gyrus (rSPG)	15	–71	55	2.84
Right anterior Supramarginal Gyrus (aSMG)	63	–24	25	3.30
Right ventral Premotor Cortex (PMv)	60	8	25	3.10
**F. Haptically-guided Grasping vs. NoGo (*Z* > 2.3, *p* = 0.05 cluster corrected)**				
Left Parietal Operculum	–55	–33	27	3.93
Right Parietal Operculum	54	–31	27	3.54
Left ventral Premotor Cortex (PMv)	–56	6	23	3.88
Right ventral Premotor Cortex (PMv)	60	9	20	3.63
Left Supplementary Motor Area (SMA)	–3	–12	51	3.99
Right Supplementary Motor Area (SMA)	0	–12	52	3.10
Left pre-Supplementary Motor Area (pre-SMA)	0	–7	51	3.16
**MAJOR CONTRASTS FROM THE LOCALIZER SCANS**				
**G. aIPS/LOtv localizer: haptic object exploration vs. passive touch (*Z* > 2.3, *p* = 0.05 cluster corrected)**				
Left Lateral Occipital Cortex tactile-visual (LOtv)	–53	–72	–5	3.09
Left anterior Intraparietal Sulcus (aIPS)	–41	–36	48	4.54
Left ventral premotor cortex (PMv)	–55	6	16	4.65
Left dorsal premotor cortex (PMd)	–26	–11	55	5.17
Left primary somatosensory cortex (SI)	–45	–28	50	4.82
Left pre-Supplementary Motor Area (pre-SMA)	–11	–4	57	5.0
Left Supramarginal Gyrus (SMG)	–59	–25	29	4.45
Left Thalamus	–14	–20	–3	4.20
Left Cerebellum	–18	–54	–31	5.27
Right dorsal premotor (PMd)	33	–11	60	4.52
Right primary somatosensory cortex (SI)	58	–21	49	4.4
Right pre-Supplementary Motor Area (pre-SMA)	7	4	53	5.87
Right Thalamus	16	–18	2	4.24
Right Cerebellum	16	–56	–32	5.33
**H. LO localizer: intact vs. scrambled objects (*Z* > 2.3, *p* = 0.05 cluster corrected)**				
Left Lateral Occipital Cortex (superior division)	–38	–69	21	4.28
Left Lateral Occipital Cortex (inferior division)	–44	–88	–1	3.20
Left Fusiform Gyrus	–34	–41	–24	3.96
**I. MT+ localizer: visual stimuli in motion vs. static stimuli (corrected voxel *p* = 0.001)**				
Left MT+	–46	–77	3	7.27
Right MT+	47	–71	2	5.79
Right Occipital Pole	8	–94	0	6.20

Average peak coordinates (in MNI space) and their peak values (Z statistics) in functional areas (or regions) identified in major contrasts from the main study, and localizer scans.

### Dorso-ventral gradients of sensitivity to object features and finger pre-shaping during haptically-guided grasping

Figure 4D shows the results of two critical comparisons involving grasping tasks mentioned above, namely the contrast of *grasping* vs. *reaching* (both tasks performed in the presence of complex objects, but requiring completely different movement kinematics), and the contrast of *grasping complex* vs. *simple objects* (with the former requiring at least increased processing of axis of elongation, and the actual object length). The difference between Figures 3D, 4A, respectively, and the overlays presented in Figure 4D is such that for obtaining the latter effects, the more laborious, but also robust, mixed-effects model, with the random-effects components of variance estimated with FSL's Flame 1+2 procedure was used for statistical analyses, hoping that it would also reveal ventral-stream contributions to these tasks. This was not the case. Interestingly, a contrast of grasping complex vs. simple objects profited much more from this approach because not only the superior parieto-frontal activity was now revealed in the right hemisphere but, additionally, it showed bilateral contributions from small subdivisions of the SMA complex and CMC. Moreover, it is quite apparent from the inspection of Figure 4D that the location of clusters showing sensitivity to object features, and sensitivity to hand preshaping is quite different, more dorsal and ventral, respectively. Yet, there is also a substantial area of overlap. Indeed, as shown by inclusive contrast masking procedure carried out in both directions for the two contrasts, all the voxels and, more importantly, only the voxels in the overlapping regions do show significant sensitivity to both object features and hand preshaping (movements kinematics) during grasp performance. In sum, the more superiorly located the area the greater sensitivity to object features during grasping, whereas the more inferiorly located the area the greater sensitivity to actual finger movement kinematics, rather than objects themselves. Of course, in the overlapping regions there is significant sensitivity both to the critical features of the grasped stimuli and the associated hand actions.

### The results of ROI analyses

The selected regions include areas significantly involved in the major tasks alone, such as object exploration—rMFG, PMv, aIPS, and grasp planning—pMTG, or to some extent involved in two tasks, e.g., exploration and grasping—TOC. Moreover, a few distinct functional subdivisions within the lateral temporo-occipital cortex were chosen for more theoretical reasons, including the human homolog of motion sensitive area MT+, a subdivision of the lateral occipital cortex sensitive both to tactile and visual processing (LOtv, revealed in our haptic aIPS/LOtv localizer), and finally the more posterior division of LO (pLO), revealed here by its exclusive sensitivity to intact vs. scrambled objects.

**Figure 5:**
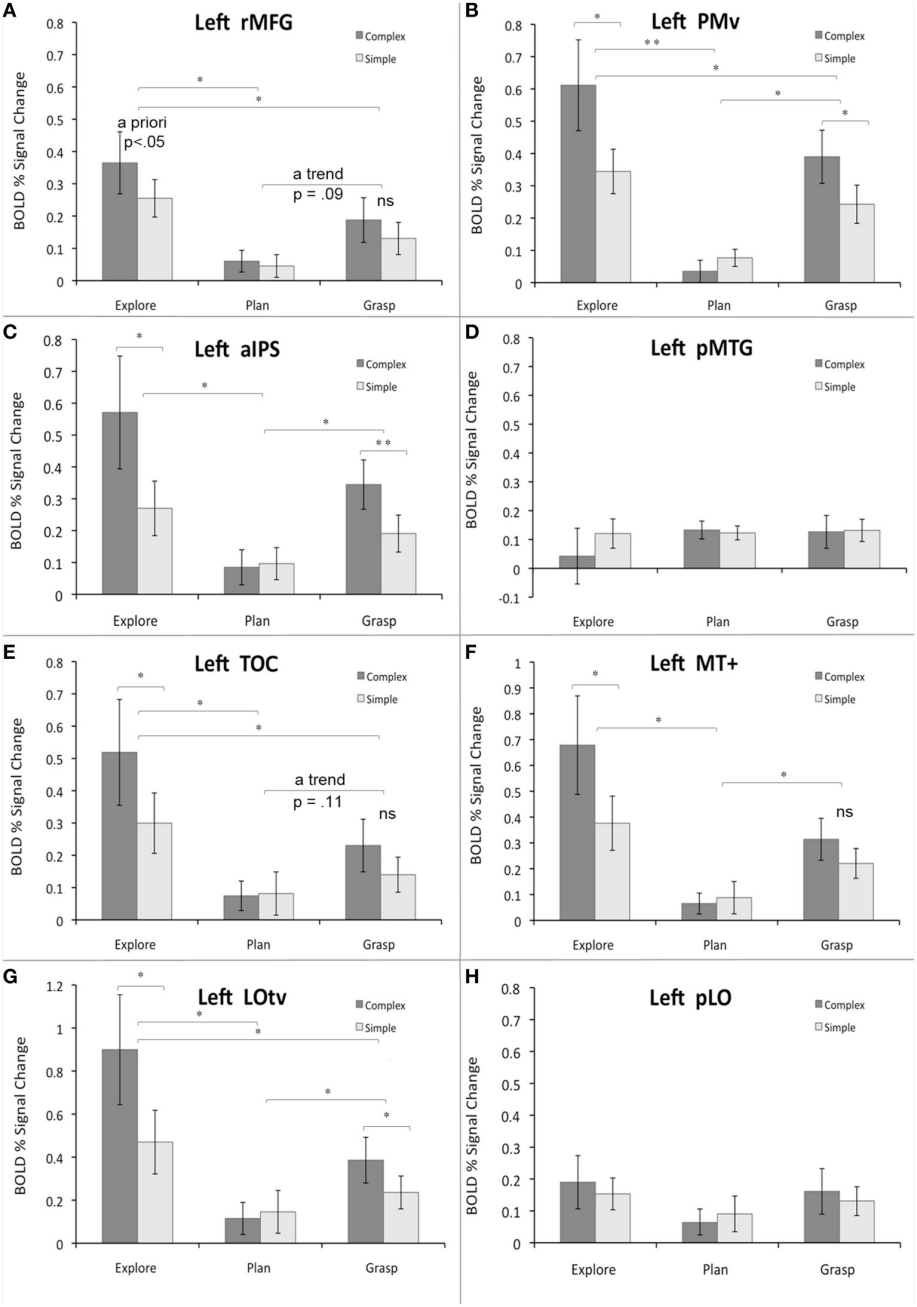
**Region-of-interest analyses for critical areas identified with different contrasts from the main study and/or localizer scans**. Panels **(A–H)** refer to specific ROIs. See main text for details. The average percent signal change within each ROI is plotted relative to resting baseline activity for the three major study phases or tasks (exploration, planning, grasping), and two object types (complex, simple). The significant main effects and simple main effects (from the significant interactions) are shown. Error bars reflect the within-subjects standard error of the mean (SEM). Asterisks indicate all the significant differences with the Bonferroni-corrected *P*-values of at least 0.05 (^*^) or 0.01 (^**^); “ns” indicates substantial but not significant differences.

In the ***rMFG* ROI**, a 3 (task) × 2 (object type) ANOVA revealed a main effect of task [*F*_(2, 18)_ = 9.3, *p* < 0.01], such that object exploration was associated with significantly stronger activity than both grasp planning, and grasp execution (Bf-p < 0.05 in both cases; whereas the grasp related activity showed only a trend toward being stronger than during grasp planning, Bf-p = 0.09). There was also a main effect of object [*F*_(1, 9)_ = 6.2, *p* < 0.05], such that performing any task involving complex objects resulted in significantly higher activity than performing the tasks with simple objects. Finally, the task by object interaction was not significant [*F*_(2, 18)_ = 1.8, *p* = 0.19]. The observed pattern of activity is shown in Figure 5A.

The ***PMv* ROI** showed a rather different pattern. Although a 3 × 2 ANOVA revealed a main effect of task [*F*_(2, 18)_ = 15.9, *p* < 0.001], such that object exploration was again associated with significantly stronger activity than both grasp planning, and grasp execution (Bf-p < 0.01, and Bf-p < 0.05, respectively), the grasp related activity was also significantly stronger than during grasp planning (Bf-p < 0.05). As above, there was a main effect of object [*F*_(1, 9)_ = 10.9, *p* < 0.01], such that performing tasks involving complex objects resulted in significantly stronger activity than performing the tasks involving simple objects. However, the task by object interaction was also significant [*F*_(2, 18)_ = 14.9, *p* < 0.001], and clearly indicated that even though exploration, and grasping of complex (vs. simple) objects did result in greater activity (Bf-p < 0.05 in both cases), this pattern was inverted for grasp planning, but the difference was not significant (Bf-p = 0.57). These results are shown in Figure 5B.

A somewhat similar pattern of activity emerged in the ***aIPS* ROI**. As in PMv, there was a main effect of task [*F*_(2, 18)_ = 7.8, *p* < 0.01], yet object exploration was linked to significantly stronger activity only when compared to grasp planning (Bf-p < 0.05), but not grasp execution (Bf-p = 0.33), with the grasp-related activity also being significantly stronger than during its planning (Bf-p < 0.05). The familiar main effect of object was revealed again [*F*_(1, 9)_ = 11.0, *p* < 0.01], with tasks involving complex objects associated with significantly higher activity than tasks involving simple objects. Finally, the task by object interaction was also significant [*F*_(2, 18)_ = 11.0, *p* < 0.001], in which only exploration, and grasping of complex objects was linked to significantly greater activity than the same tasks performed with simple objects (Bf-p < 0.05, and Bf-p < 0.01, respectively), whereas there was no object related difference whatsoever in the activity associated with grasp planning (Bf-p = 0.63). The observed effects are shown in Figure 5C.

The ***pMTG* ROI** activity pattern was among the least expected. Neither a main effect of task [*F*_(2, 18)_ = 0.39, *p* = 0.69], nor object type [*F*_(1, 9)_ = 0.92, *p* = 0.36] was significant. So was not the task by object interaction [*F*_(2, 18)_ = 1.79, *p* = 0.19]. This result is displayed in Figure 5D.

This means that only when tested in isolation, the grasp planning activity vs. rest was significantly different from baseline.

In the ***TOC* ROI** the pattern of activity was quite similar to the one observed in PMv. A main effect of task [*F*_(2, 18)_ = 10.8, *p* < 0.001] was observed, in which object exploration was associated with significantly stronger activity than both grasp planning, and grasp execution (Bf-p < 0.05 in both cases), but despite a trend, grasp execution did not engage this area more than grasp planning (Bf-p = 0.11). A main effect of object [*F*_(1, 9)_ = 10.9, *p* < 0.01] was also significant and, again, performing tasks involving complex objects was associated with higher activity than performing the tasks involving simple objects. Finally, the task by object interaction was also significant [*F*_(2, 18)_ = 6.9, *p* < 0.01], but now only the exploration of complex objects resulted in significantly higher activity than exploration of simple objects (Bf-p < 0.05), with no difference between objects whatsoever for grasp planning (Bf-p = 1.0), and even lack of substantial trend for grasping complex vs. simple objects (Bf-p = 0.25). The results are shown in Figure 5E.

In the more posterior ***MT+* ROI** the pattern of activity resembled that of aIPS. The analysis showed a main effect of task [*F*_(2, 18)_ = 10.0, *p* < 0.001] such that only object exploration, and grasp execution, was associated with significantly stronger activity when compared to grasp planning (Bf-p < 0.05 in both cases), whereas exploration and grasp execution activity did not differ (Bf-p = 0.19). The familiar main effect of object was revealed as well [*F*_(1, 9)_ = 10.5, *p* < 0.01], where tasks involving complex objects were associated with significantly stronger activity than tasks involving simple objects. Finally, there was also a significant task by object interaction [*F*_(2, 18)_ = 4.4, *p* < 0.05], yet only exploration of complex objects was linked to significantly greater activity than exploration of simple objects (Bf-p = 0.05), whereas the effect was absent for grasp planning (Bf-p = 1.0), and almost non-existent for grasp execution (Bf-p L = 0.24). These changes in activity are shown in Figure 5F.

The pattern was nearly the same for the ***LOtv* ROI**. A main effect of task was again significant [*F*_(2, 18)_ = 11.5, *p* < 0.001], wherein object exploration, and grasp execution, was associated with significantly stronger activity than grasp planning (Bf-p < 0.05 in both cases), but exploration also invoked stronger activity than grasp execution (Bf-p = 0.05). The main effect of object was revealed again [*F*_(1, 9)_ = 15.1, *p* < 0.01], wherein tasks involving complex objects invoked significantly higher activity than simple objects. Finally, a significant task by object interaction [*F*_(2, 18)_ = 10.1, *p* < 0.001] was such that both exploration and grasping of complex objects was linked to significantly stronger activity than exploration and grasping of simple objects (Bf-p = 0.05 in both cases), whereas the effect was absent for grasp planning (Bf-p = 1.0). The observed pattern of activity is shown in Figure 5G.

Finally, in the ***pLO* ROI**, similarly to *pMTG* ROI, neither a main effect of task [*F*_(2, 18)_ = 1.4, *p* = 0.27], nor object type [*F*_(1, 9)_ = 0.5, *p* = 0.5] was significant. Similarly, there was no significant task by object interaction [*F*_(2, 18)_ = 0.86, *p* = 0.44]. The result can be seen in Figure 5H.

## Discussion

In this study, to our knowledge, for the first time, we examined the patterns of neural activity associated with grasping of complex objects that do not have any prior visual representations in the brain. To this end, participants first explored the novel targets haptically in order to determine their shapes and orientations, planned grasping these objects a couple of seconds later, and following a short variable interval, executed the pre-planned grasps.

The activity associated with haptic exploration of the targets included the fronto-parietal, temporo-occipital, and insular cortices (Binkofski et al., 1999a; Deibert et al., 1999; James et al., 2002; Sathian, 2005). Interestingly, given the ultimate goal of the exploratory phase, i.e., preparation for later grasping, the engaged networks comprised of all the areas commonly associated with *the praxis representation network* or PRN (e.g., Frey, 2008; Króliczak and Frey, 2009; see also Króliczak et al., 2008; cf. Snow et al., 2015). Yet, the observed signal changes were less left lateralized and the clusters devoted significantly to the processing of object shape were lateralized exclusively to the right hemisphere. Indeed, they were restricted primarily to the anterior and rostral divisions of the posterior parietal cortex.

To our surprise, although the areas involved in grasp planning belonged largely to the left hemisphere, there was almost no overlap with those involved in object exploration. Namely, the temporal clusters were more anterior, and the single intraparietal cluster was more posterior and, even more unexpectedly, none of them showed any sensitivity to object features (cf. Valyear et al., 2007; Glover et al., 2012; Singhal et al., 2013).

Even though the networks re-recruited for grasp execution were similar to those involved in object exploration, only the *dorso-dorsal* (Rizzolatti and Matelli, 2003) and predominantly bilateral parieto-frontal networks showed clear modulations depending on object complexity (cf. Binkofski et al., 1999a; Reed et al., 2005). These networks included nearly all the right-hemisphere voxels that revealed object sensitivity in the exploration phase. The region of interest analyses further corroborated these results, demonstrating—as in the study by Króliczak et al. (2007)—no task/object selectivity during grasp performance in areas typically associated with *visual perceptual processing* of shape and size, or object affordances, such as the lateral occipital or TOC (e.g., James et al., 2003; Vingerhoets, 2008).

Because neither the visual nor *multimodal* perceptual, ventral-stream regions showed any pronounced sensitivity to object shape and orientation in the separate phases of the paradigm used here, similarly to visually-guided actions (e.g., Culham et al., 2003; Króliczak et al., 2008; for a recent review see Gallivan and Culham, 2015) their contribution to the haptic control of grasping is marginal at best. Analogous conclusions can be also drawn about the involvement of the insular, and secondary somatosensory cortices. While greater engagement of selected ventral ROIs was observed during processing of complex shapes, most of the time these effects were quite weak and task independent (i.e., required collapsing across the exploratory and grasp phases). Instead, this study demonstrates that the critical haptic processing of object features for future manual actions takes place primarily in the right superior parietal lobule, and extends via aIPS to aSMG of the inferior parietal lobule (cf. Binkofski et al., 1999a). All these areas get re-recruited for grasp performance, and their input is dispensed bilaterally, with the inclusion of the contralateral left aIPS (and to some extent the interconnected left PMv ROI), and utilized by the parieto-frontal networks for haptic grasp guidance.

As such, these results suggest that a substantial portion of what has been taken as evidence for visual perceptual processing in the parietal lobe can reflect primarily a conversion from visual—object-related processing of shape for action—to haptic codes for the on-line control of the grasping hand (cf. Cohen and Andersen, 2002; see also Culham et al., 2006). A clear support for this proposal comes from our observation of the *dorso-ventral gradients of haptic sensitivity* to object features and finger pre-shaping, respectively, during haptically guided grasping. The area most commonly studied in the context of grasp performance, namely aIPS, is located somewhere in the middle of this gradient, and shows significant sensitivity to both object characteristics and the required kinematics (even though the targets were never seen before).

### Haptic object exploration involves the use of praxis skills, and visual encoding

In addition to the major nodes of the PRN, the activity associated with haptic exploration of the novel objects involved both the medial and lateral occipital cortices, and the more anterior, mainly lateral temporo-occipital regions. Such a pattern of results is not surprising given that, with their exploratory finger movements, participants were to look for object features that were most *diagnostic* (i.e., characteristic/important) for later performance of the grasping task, and these features could arguably be shape and orientation. Moreover, the use of such skills as executing initial exploratory “grasp-type” enclosure on an unknown target, dynamic molding to and/or following of its contours, and finding the axis of elongation does not only permit efficient extraction of an object form but is typically associated with the *visual encoding bias* (Lederman et al., 1996). Indeed, as shown by Lederman *et al.*, such a bias is even more likely when variations in shape are more *perceptually accessible* than any other properties of the studied objects. This was definitely the case for the novel stimuli used in our study. Thus, the simultaneous engagement of PRN and visual regions during haptic exploration is consistent with the use of the grasp-like, and contour-tracking exploratory strategies, and the closely associated inclination for visual encoding.

It was rather unexpected, though, that this kind of processing would not result in more wide-spread differential signal changes reflecting object complexity (cf. Valyear et al., 2006 for the visual modality). Yet, the ultimate goal of the exploratory movements was a preparation for grasping, not object discrimination (or recognition), and encoding of all the details related to object shape was not even necessary. Indeed, the prerequisite of skilled grasping in this paradigm was finding the orientation of the object—basically its axis of elongation, and then encoding its extent (*length*) along this particular dimension. Similarly, the thorough coding of perceptual properties of objects, and their relations to other targets, could have been disregarded (cf. Hesse et al., 2008).

The less focus on haptic processing of details, the less differential object-related activity would be expected in the left hemisphere, particularly in the ventral stream. Conversely, the crude or more global haptic processing of shape (e.g., finding only appropriate “grasp points”) is expected to involve the right superior parietal lobule (Dijkerman and de Haan, 2007; e.g., Leisman and Melillo, 2007; see also Milner and Goodale, 1995). Consistent with this notion is an observation that only a small LOtv cluster, as well the more posterior subdivision of the left TOC, namely MT+, exhibited some selectivity to object shape in the ROI analyses (For LOtv it was found both during exploration and grasping, and for MT+ only during object exploration, resulting in a similar effect in the whole TOC ROI for object exploration). Yet, such sensitivity was not revealed by any of the whole brain contrasts from the main study. Moreover, this kind of response selectivity could be also accounted for by some tuning of this area to more complex patterns of exploratory finger movements, rather than shape processing *per se* (cf. Amedi et al., 2002; see also Lederman et al., 1996).

In sum, the putative visual encoding bias in haptic exploration of object contour and extent has been insufficient for generating reliable and wide spread object-shape sensitivity in the ventral processing stream. Although the results of ROI analyses indicate that object selectivity may nevertheless be found in subdivisions of the lateral and ventral temporo-occipital cortex, it can be associated with multimodal interactions of shape. That is, it can be linked to moment-to-moment finger postures, or even to monitoring of the more complex finger movements during contour tracking. Only the more anterior, and often rostral divisions of the right posterior parietal lobe have shown indisputable haptic shape/orientation sensitivity (cf. Binkofski et al., 1999a; James et al., 2002; Reed et al., 2005; Dijkerman and de Haan, 2007), and these regions most likely provide the critical input for the execution of later grasping.

### Haptically-guided grasp planning does not invoke regions sensitive to visual object shape

To our surprise, unlike in visually-guided delayed grasping, where specialized dorsal-stream areas contribute both to planning of action and maintenance of its goals (e.g., Singhal et al., 2013), none of the *dorso-dorsal* nor *ventro-dorsal* networks (Rizzolatti and Matelli, 2003) were re-recruited here during the planning phase. Yet, consistent with the idea that in the ventral stream of information flow the inputs pass through progressively more complex stages of processing (resulting in global object representations linked to memory), during the grasp planning phase we observed significant recruitment of the left lateral and ventral temporal cortices anterior to the TOC region engaged in object exploration. This outcome is also in agreement with a notion that the lateral occipito-temporal cortices appear to be less task-specialized and may play associative functional roles (e.g., Monaco et al., 2014), particularly during action planning rather that its execution (for a review, see Króliczak et al., 2012). Even more importantly, though, grasp planning under haptic guidance has been also associated with sustained bilateral signal in EVCs (particularly with relatively early visual cortex signal modulations; see Singhal et al., 2013). Although this effect is consistent with the employment of the *visual encoding bias* (Lederman et al., 1996), it must reflect some pretty basic “visualization skills” because only the more posterior medial and ventral occipital areas have shown any overlap with those involved in object exploration.

It is of particular note that neither the areas with the sustained, nor the ones with newly induced significant signal changes showed any object shape selectivity (cf. Valyear et al., 2007; Glover et al., 2012; Singhal et al., 2013), a finding that was also corroborated by the less stringent ROI analyses. Indeed, with the exception of left pMTG, the observed signal changes were characteristically very small (<0.1% of BOLD signal change) and, oftentimes, showed activity patterns going in the direction opposite to neural responses typically observed for complex vs. simple objects. Thus, even though the observed signal modulations may reflect some *preparatory set* activity (cf. Connolly et al., 2002; Valyear and Frey, 2015), it is quite unlikely that its role is to uniquely link the parieto-frontal grasp networks with the temporo-occipital visual/multimodal areas (cf. Borra et al., 2008; see also Króliczak et al., 2008). This activity may nevertheless play an important role in the later re-recruitment of the parieto-frontal networks for the proper grasp type and hand orientation.

### Haptically-guided grasping is associated with fMRI activity in dorsal but not ventral stream brain areas

Counter to grasp planning, the actual execution of the grasp based on the haptic information obtained a few seconds before was accompanied by re-recruitment of areas associated with haptic object encoding, and extensive bilateral engagement of the dorsal, parieto-frontal networks. While in the haptic domain this outcome does not necessarily indicate a dissociation between perceptual- and action-related processing (cf. Binkofski et al., 1999b; Reed et al., 2005; see also Whitwell et al., 2014; and Whitwell et al., 2015), it is inconsistent with the notion that the control of manual actions requires both dorsal and ventral stream contributions (e.g., as in delayed visually-guided actions; Singhal et al., 2013).

It is worth emphasizing that the ventral-stream contribution to grasping was likely to occur (when compared to resting baseline) given the observed engagement of EVCs during haptic exploration. Yet, while pointing to the use of the representations based on the preceding visual encoding bias (Lederman et al., 1996; cf. Amedi et al., 2002), neither the EVCs nor the more anterior temporal regions showed any object shape sensitivity. Therefore, their contribution to grasp guidance could have been only of a very general nature. Indeed, this study revealed fast and substantial decay of fMRI activity in temporo-occipital regions when object exploration was complete. Notably, the re-recruitment of their more specialized subdivisions (i.e., LOtv, MT+) was revealed neither in the planning nor the execution phase in the whole brain analyses. Although, some sensitivity to object features was identified in the left LOtv ROI during grasp-execution, it more likely reflects the control of the on-going finger preshaping and the ultimate grasp enclosure on the shaped object contour, rather than the overall perceived object shape.

The lack of substantial ventral contribution to haptically-guided grasping is not that surprising because even in studies on visually-guided actions it is quite controversial whether or not the ventrally encoded *perceptual representations* are used for the guidance of the grasping hand (e.g., Cavina-Pratesi et al., 2007; Króliczak et al., 2007; cf. Króliczak et al., 2008). Indeed, a large body of evidence suggests that, at least in the case of hand movements directed at simple targets, the remembered (ventrally-encoded) information on object shape, size and/or its relative location seem to play a marginal role when the planning and/or execution of actions takes place in full vision (e.g., Monaco et al., 2010, 2015; see also Culham et al., 2003; Króliczak et al., 2006; cf. Hesse and Franz, 2009; Valyear and Frey, 2015; for a review see Goodale et al., 2005; Króliczak et al., 2012). Although such *on-line* action guidance can be typically handled almost exclusively by the *dorsal*, visuo-motor processing stream (Goodale and Milner, 1992; Milner and Goodale, 2008; see also Goodale et al., 2008; Goodale, 2014), when grasp planning and/or its execution is only briefly delayed, or vision is fairly limited, the visuo-motor system can hardly operate without such stored visual input (e.g., Goodale et al., 1994; Milner et al., 2001; Westwood and Goodale, 2003; see also Monaco et al., 2010; see also Whitwell et al., 2014, 2015). For the same reasons, when substantially longer delays are introduced after object viewing the re-recruitment of areas in the visual perceptual stream becomes even more essential for the performance of grasping actions (Singhal et al., 2013). Yet, whatever mechanisms are involved in the guidance of the grasping hand based on previous visual input, they are clearly less relevant when grasp performance is based entirely on the just acquired, and transiently stored, haptic input.

Of utmost importance is the observation that the re-recruited lateral and medial *dorso-dorsal* (Rizzolatti and Matelli, 2003) parieto-frontal networks of the left hemisphere did not show any sensitivity to object features during earlier phases. Yet, during grasp performance nearly all their critical nodes, such as adSPOC, rSPG, PMd, SMA, and CMC already operated on such representations. Thus, although one of the roles of the dorso-dorsal stream might be the provision of inputs for the comparison of the somatosensory/proprioceptive feedback with a forward model of the programmed grasp (cf. Makoshi et al., 2011; Singhal et al., 2013), in our opinion such a role cannot be effectively fulfilled in the absence of object coding performed elsewhere. This hypothesis is based on our findings that the source of object sensitivity based exclusively on haptic information was in fact located predominantly in the anterior right PPC. Yet, regardless of the origin of this sensitivity, all the aforementioned dorso-dorsal areas have been shown capable of processing the most critical object dimensions, and providing or even transforming their input to action codes for the ventro-dorsal areas that control the on-going grasp movement kinematics.

Our outcomes clearly demonstrate that the *dorso-dorsal* parietal and frontal areas of the left hemisphere show greater sensitivity to object features, whereas the *ventro-dorsal* parietal areas are more sensitive to the actual grasp kinematics. Of course, left aIPS is capable of processing relevant target features and selecting and/or monitoring action kinematics both under visual (Króliczak et al., 2008) and haptic guidance. The overall gradients of activity within the right hemisphere, including aSMG were quite similar, including the medial dorso-dorsal divisions and their projections to the frontal cortex (Rizzolatti and Matelli, 2003). Yet, judging by the overall distribution of this activity, it seems that the right hemisphere parietal areas might be more involved in the comparisons of the somatosensory and proprioceptive feedback with predicted movement plans. It should be re-iterated, though, that the haptic sensitivity to object features has its source here, and during haptically-guided grasping is not only retained but also extends to functional areas that were not involved in—or perhaps were even actively suppressed during—object exploration (cf. Binkofski et al., 1999a; Reed et al., 2005).

Given how efficiently the localized, right-hemisphere activity can be distributed across the bilateral parieto-frontal networks, the outcomes of our study also shed a new light on the mechanisms involved in ego-centric coordinate transformations taking place in the parietal lobe (Cohen and Andersen, 2002; Milner and Goodale, 2008). Indeed, instead of representing purely visual transformations between different frames of reference, the parietal lobe activity can often reflect primarily a conversion from visual, haptic and/or modality-independent object coding to (egocentric) haptic and even kinesthetic codes for the control of the grasping hand (cf. Whitwell et al., 2014; Leoné et al., 2015; Whitwell et al., 2015).

### Limitations of the study

One of the potential limitations is the lack of a visual control task. Yet, haptic exploration typically takes time, while visual exploration would be effective almost instantaneously. Moreover, if we used a control task that would require later object discrimination (or even its recognition), perhaps more areas sensitive to object complexity would be revealed, especially in the ventral stream. In terms of timing, if the exploration phase was shorter, which would also make it harder, and subsequent phases were delayed in time, more clear-cut differences in activity could emerge for complex vs. simple objects. It is also of note that our participants were required to explore the circular disks for the entire 5 s, which could in some areas result in steady increases of the signal, leading eventually to its saturation and/or ceiling effects. Yet, purely perceptual haptic areas should respond less for circular disks due to adaptation following repeated movements over the same shape. Furthermore, perhaps the outcomes from the planning phase would be more intuitive if grasp planning was separated from the exploration phase by a much longer interval. Finally, the paradigm could profit a lot from a clear distinction between grasping and reaching trials made up front. That is, if participants knew right from the beginning of a trial that they were to reach, and thought about moving the clenched fist toward the target instead, the outcome of the comparison with grasping could likely be even more informative.

## Conclusions

While the importance of vision for action planning and execution has long been recognized (Helmholtz, 1867/1962), the role of the haptic sense for critical daily interactions with objects has received considerably less attention. While this gap has been recently narrowed (Dijkerman and de Haan, 2007) and more ecologically valid paradigms are being used and considered (Stone and Gonzalez, 2015), there is still a substantial work to be done in the domain of tactile processing and haptic perception (cf. Snow et al., 2015), as well as in the area of haptically-guided actions. Although this study did not take into account common objects, all the phases that are essential for such interactions were investigated here. We found that the most critical aspects of task performance are controlled by the dorsal-stream regions, with much greater—in fact exclusive—contribution of the right hemisphere to the haptic processing of object shape (or its exact graspable dimension), and the bilateral involvement of the parieto-frontal networks, including left aIPS, in the control of the haptically-guided grasping. Furthermore, two different kinds of signal processing for grasp performance have been associated with the *dorso-dorsal* vs. *ventro-dorsal* parieto-frontal networks (thus forming a dorso-ventral gradient), with the emphasis on representing grasp-relevant features of the unseen targets, and monitoring of grasp kinematics, respectively. Of course, intermediate areas such as aIPS show sensitivity to both object shape and the required grip kinematics. Finally, these outcomes suggest that the transformations for action guidance in the parietal lobe reflect primarily re-coding of object-related into effector-related representations, regardless of the sensory input modality, and independent of the ventral stream. Although there is substantial evidence that in more cognitive tasks haptic memory is supported by dedicated ventral somatosensory-insular-(pre)frontal cortex pathways (e.g., Burton and Sinclair, 2000; Pasternak and Greenlee, 2005), the role of these and other ventrally-located regions in tasks and paradigms similar to ours will likely be limited to supportive or associative functions (cf. Bidula and Kroliczak, 2015).

## Author contributions

This project was conceptualized by GK and MM. Data was collected by GK, analyzed by GK, MM, AK, and interpreted by all the authors. The manuscript was written by GK, MM, and AK.

### Conflict of interest statement

The authors declare that the research was conducted in the absence of any commercial or financial relationships that could be construed as a potential conflict of interest.

## Supplementary material

Supplementary Figure 1 **All the objects, their orders, and orientations used in the main experiment**. Note, that there were five runs for each subject and one of the sets was repeated.

Supplementary Figure 2 **Neural activity associated with haptic exploration of simple vs. complex objects**. This contrast revealed modulations in a widespread network of areas, resembling the default mode network.
