# Supplementary figures and images for: Haptically Guided Grasping. fMRI Shows Right-Hemisphere Parietal Stimulus Encoding, and Bilateral Dorso-Ventral Parietal Gradients of Object- and Action-Related Processing during Grasp Execution
Source: Front Hum Neurosci. 2016 Jan 5;9:691. doi: 10.3389/fnhum.2015.00691 (PMC4700263; doi:10.3389/fnhum.2015.00691)

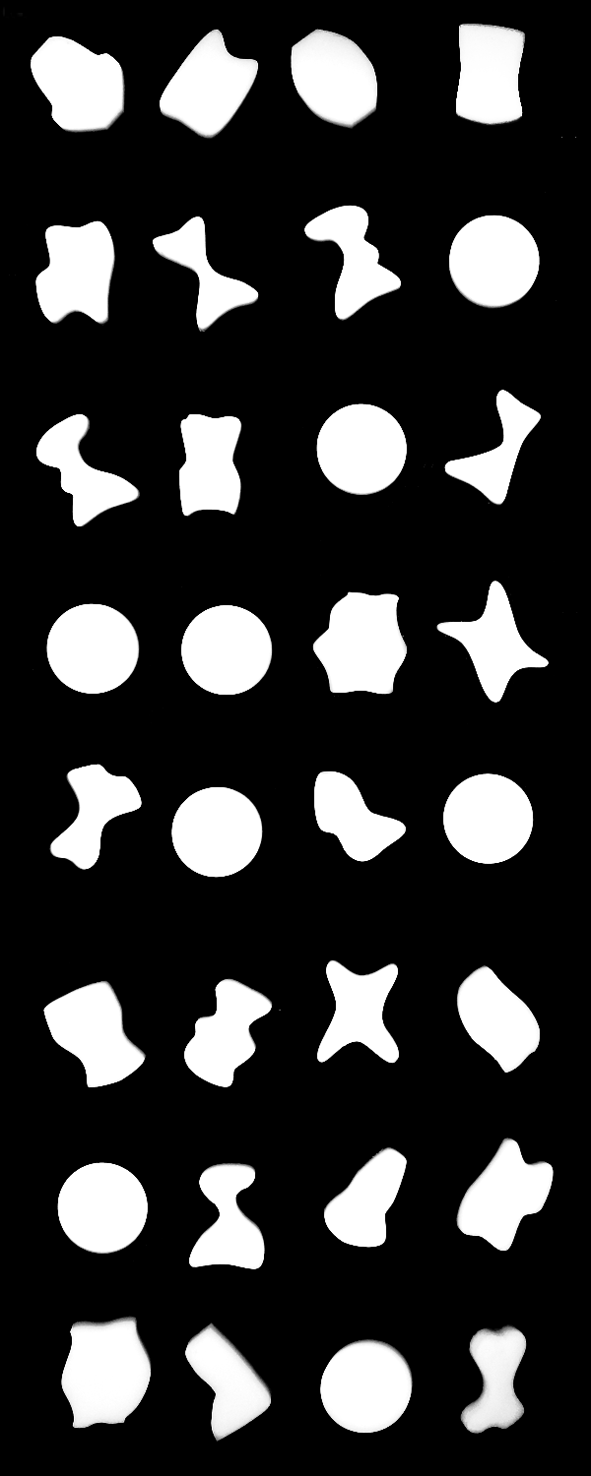

Supplement: Supplementary Figure 1 — All the objects, their orders, and orientations used in the main experiment. Note, that there were five runs for each subject and one of the sets was repeated. [file Image1.TIFF]

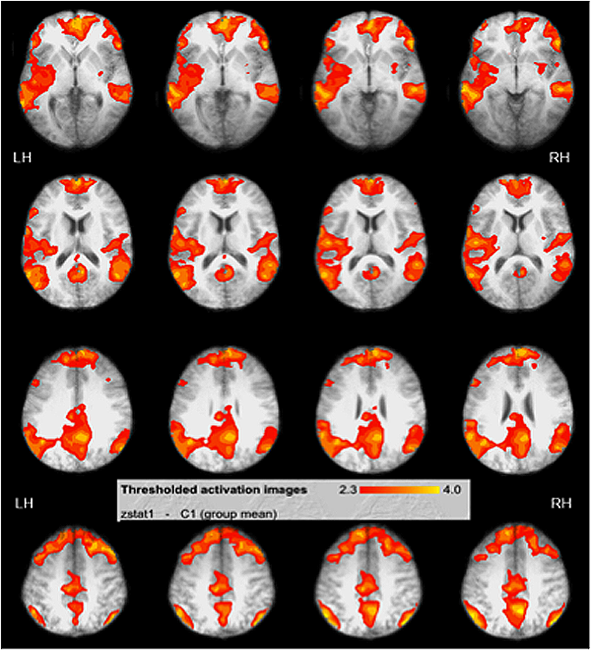

Supplement: Supplementary Figure 2 — Neural activity associated with haptic exploration of simple vs. complex objects. This contrast revealed modulations in a widespread network of areas, resembling the default mode network. [file Image2.TIFF]
